# In Memoriam: Professor Jarosław Lewkowski (1966–2019)

**DOI:** 10.3390/ma13184030

**Published:** 2020-09-11

**Authors:** Grzegorz Mlostoń, Jarosław Romański

**Affiliations:** Department of Organic and Applied Chemistry, University of Lodz, Tamka 12, 91-403 Łódź, Poland


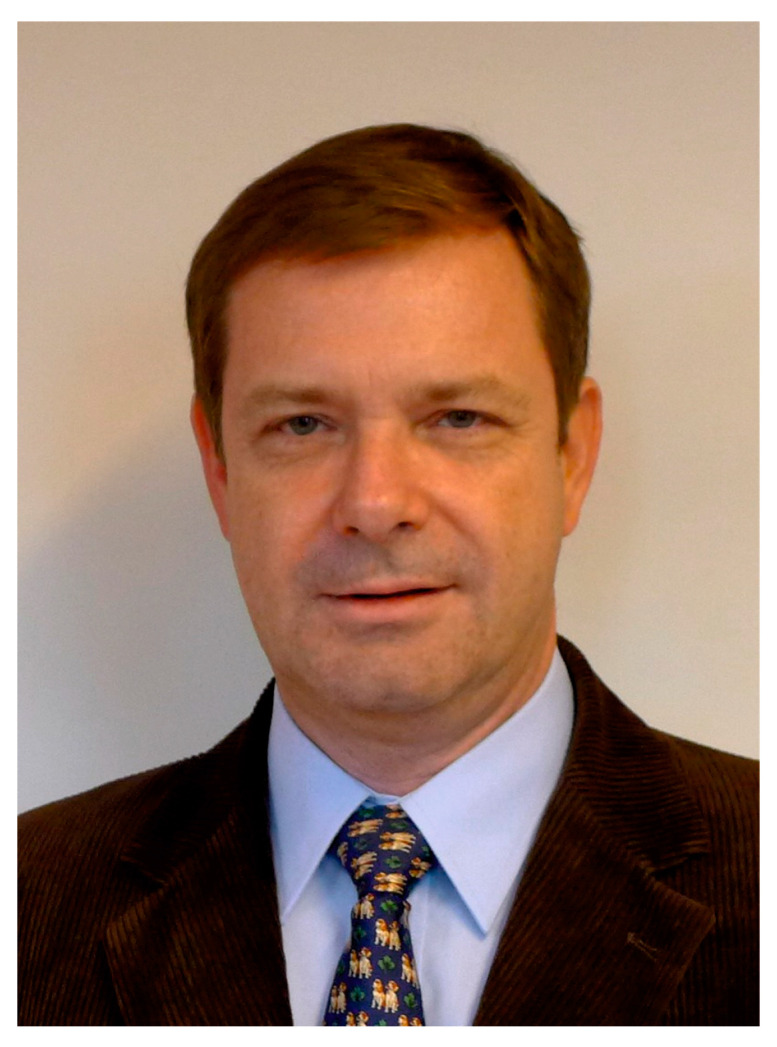


We are deeply saddened after the unexpected and untimely death of Jarosław Lewkowski, (Faculty of Chemistry, University of Łódź, Poland) who died at the age of 53 years on 29 November 2019. His death resulted from a heart attack, which he suffered in August just after returning from summer vacation spent on the seaside. A few months earlier, in April 2019, Jarosław received the Professor title from the President of the Republic of Poland, Andrzej Duda. For almost one year we had been working jointly in a three-membered team on a thematic issue of Materials, entitled: Current Problems of the Organic Chemistry of Sulfur and Selenium. He collaborated very well and served actively as an invited co-editor. In collaboration with a group of his associates from Jan Długosz University in Czestochowa, he submitted an excellent—and for him final—manuscript which appeared as the first contribution to this issue.

Jarosław was born and educated in Łódź. The PhD thesis entitled ‘Selektywne przemiany furfuralu, 5-hydroksymetylofurfuralu oraz ich pochodnych’ (Selective conversions of furfural, 5-hydroxymethylfurfural and their derivatives) (1996) was prepared at the University of Łódź under the supervision of Professor Romuald Skowroński, his academic teacher and scientific mentor. After several postdoctoral stays at French Universities in Lyon (group of G. Descotes), Montpellier (groups of H.-J. Cristau and J.-L. Pirat), Toulouse (group of L. Rigal), and Rennes (groups of J. Mortier and M. Vaultier) he returned to Łódź in 2001 and started to prepare the Habilitation Thesis, which was finished in 2006 and successfully defended at the home University. From this year on, Jarosław built up and then chaired his research group located at the Department of Organic Chemistry. The main field of his scientific interest covered problems of experimental heterocyclic and hetero-organic chemistry, with the focus on the organic chemistry of phosphorus, selenium, and sulfur. Jarosław used to work actively for the scientific community, and over two periods between 2013 and 2018 occupied the position of the chairman of the local branch of the Polish Chemical Society in Łódź, and in the last two years of his life he was an elected member of the Executive Committee of the Łódź Scientific Society as well. He was a warm and generous academic teacher, well known and beloved by students not only at the Chemistry Faculty but also at other faculties of the University of Łódź, where he lectured on history and general importance of chemical sciences in modern societies over years.

We will be recollecting his good judgment, unique, but very pleasant sense of humor as well as devotion to high quality of the research work, which he frequently demonstrated as an academic teacher and reviewer of evaluated manuscripts. For these reasons he was nominated to the editorial boards of several journals, and the last nomination to the editorial board of *Phosphorus, Sulfur, Silicon and Related Elements* he received at the board meeting held in Rostock, in July 2019. As a Faculty member, Jarosław was always a loyal colleague and friend, ready to offer nonprofit help and advice in many instances. Therefore, he will be remembered by many people who knew him with great respect and gratitude for a long time; at the University of Łódź, we are missing Jarek very much.

